# Centrifugation does not remove bacteria from the fat fraction of human milk

**DOI:** 10.1038/s41598-020-79793-y

**Published:** 2021-01-12

**Authors:** Lisa F. Stinson, Jie Ma, Alethea Rea, Michael Dymock, Donna T. Geddes

**Affiliations:** 1 School of Molecular Sciences, The University of Western Australia, Perth, Australia; 2 Mathematics and Statistics, Murdoch University, Perth, Australia; 3 Centre for Applied Statistics, The University of Western Australia, Perth, Australia

**Keywords:** Bacteria, Microbial communities, Biological techniques, Microbiology, Medical research

## Abstract

Analysis of the human milk microbiome is complicated by the presence of a variable quantity of fat. The fat fraction of human milk is typically discarded prior to analysis. It is assumed that all cells are pelleted out of human milk by high speed centrifugation; however, studies of bovine milk have reported that bacteria may remain trapped within the fat fraction. Here, the bacterial DNA profiles of the fat fraction and cell pellet of human milk (n = 10) were analysed. Human and bacterial DNA was consistently recovered from the fat fraction of human milk (average of 12.4% and 32.7%, respectively). Two low-abundance *Staphylococcus* species (< 0.5% relative abundance) was significantly more abundant in the cell pellet compared to the fat fraction (*P* < 0.04), and three low-abundance species (< 5% relative abundance) were recovered from one fraction only. However, inclusion of fat reduced the efficiency of DNA extraction by 39%. Culture-based methods were used to quantify the distribution of an exogenously added strain of *Staphylococcus aureus* in human milk fractions. *S. aureus* was consistently recovered from the fat fraction (average 28.9%). Bacterial DNA profiles generated from skim milk or cell pellets are not representative of the entire human milk microbiome. These data have critical implications for the design of future work in this field.

## Introduction

Human milk contains a low biomass of bacteria^1^ which contribute to the seeding of the infant oral and gut microbiomes^2–6^. Research interest in the human milk microbiome is growing; however, there are a number of methodological challenges in this field^7^. While 16S rRNA gene sequencing studies have identified a “core” human milk microbiome consisting of *Streptococcus* spp. and *Staphylococcus* spp., the overall bacterial profile of human milk varies significantly between studies. Variation may due to individual host conditions, such as geographical location^8^, diet^9^, or health conditions^10^. However, it may also be reflective of biases introduced during sampling, DNA extraction, PCR amplification, and data analysis^11^.

The fat fraction of human milk presents a technical challenge for microbiome research. Milk fat can interfere with DNA extraction methods^12^, for instance by blocking spin column filters, and can act as a PCR inhibitor^13^. For these reasons, human milk samples are routinely centrifuged prior to DNA extraction and the fat layer is discarded^1,8,14–20^. It is assumed that all cells in human milk are pelleted out upon high speed centrifugation, as they are for other sample types; however, evidence from bovine studies suggests that bacteria may be trapped within the fat fraction^21,22^. Sun et al. spiked three bacterial species, *Staphylococcus aureus*, *Escherichia coli*, and *Lactobacillus reuteri*, into whole bovine milk, then fractionated each sample^21^. While the majority of the bacteria separated out to the pellet (73.5–92.6%), a proportion remained in the fat layer (7.4–26.5%). Using fluorescent in situ hybridisation, it was found that *L. reuteri* R2LC was associated with milk fat globules in whole milk. Interestingly, their images appear to demonstrate that this bacteria adheres to the outer membrane of milk fat globules. In a similar study, Fijałkowski et al*.* spiked a low inoculum of a mastitis-causing *S. aureus* strain into whole and skim bovine milk^23^. The growth of this strain in skim milk was approximately half of that of whole milk. These results are supported by 16S rRNA gene sequencing studies, which demonstrate that bovine milk fat not only contains bacterial DNA, but that the bacterial DNA profiles in this fraction can vary from those of the skim fraction (cell pellet and supernatant)^21,22^.

Collectively, these studies suggest that bacterial cells are not able to be completely centrifuged out of milk fat. If this also holds true for human milk, then previous studies of the human milk microbiome are likely incomplete. Another consideration is that the fat concentration in human milk varies depending on the degree of fullness of the breast, so that milk sampled at the beginning of a feed contains a lower concentration of fat than milk sampled at the end of a feed^24^. If the human milk microbiome varies according to the concentration of fat, then sampling protocols may need to be standardised based on fat concentrations. This study aimed to assess the extent to which bacteria are present in the fat fraction of human milk, and whether the bacterial profiles of human milk fat vary from those of the cell pellet.

## Methods

### Sample collection and quantification of fat content

Milk samples were collected from lactating women (1–6 months post-partum) who attended study sessions at The University of Western Australia. This study was approved by the University of Western Australia’s Human Research Ethics Committee (RA/4/1/2369) and all participants provided informed consent. All methods were carried out in accordance with relevant guidelines and regulations. For the comparison of the fat fraction and cell pellet, post-feed samples (2–3 ml, n = 10) were hand-expressed into sterile tubes after the breast had been emptied by 15 min of pump-expression using a Symphony electric breast pump (Medela AG, Baar, Switzerland). For the comparison of pre-feed (low-fat) and post-feed (high-fat) samples, a subset of mothers (n = 6) were asked to hand-express samples into sterile tubes before and after a 15 min pump-expression (2–3 ml each). For the spike-in assay, post-feed samples (2–3 ml, n = 10) were hand-expressed into sterile tubes after the breast had been emptied by 15 min of pump-expression. The fat content of each sample was measured using the Creamatocrit method after collection^25^. All samples were stored at − 20 °C until analysis.

### Fractionation and DNA extraction

Preliminary testing was performed to assess the best method for fractionation of the samples. Centrifugation was performed at 5000, 10,000, 20,000, and 40,000*g.* The firmness of the fat fraction increased at higher centrifugation speeds, and the layer could best be handled at 10,000*g.* Given that there was no statistical difference in the quantity of total DNA based on centrifugation speed, 10,000 g was used for centrifugation here.

Each sample was mixed thoroughly then divided into three 1 ml aliquots: one for the fat fraction, one for the cell pellet, and one for “whole” milk. All samples were centrifuged at 10,000*g* for 10 min at 4 °C prior to analysis to separate the fat and cell pellet. For the fat sample, the fat layer was gently moved to the side of the tube using a pipette tip, and the cell pellet and supernatant were removed by pipetting, leaving behind the fat only. For the cell pellet sample, the fat layer was first removed by scraping with a sterile culture loop. The supernatant and any remaining fat was then removed by pipetting, leaving the cell pellet only. For “whole” milk samples, the fat layer was gently pushed to the side of the tube using a pipette tip, and the supernatant was removed by pipetting, leaving both the fat layer and cell pellet. It was not possible to use a single aliquot for all samples given the physical properties of milk lipids. The fat fraction tended to stick to the outside and inside of pipette tips, and could therefore not be moved to a new tube.

Each fraction was resuspended in lysis buffer and DNA was extracted using the QIAGEN MagAttract Microbial DNA kit on the King Fisher Duo platform according to the manufacturer’s instructions. Eluates were stored at − 20 °C until analysis.

### Quantification of total, human, and bacterial DNA

Human DNA levels were quantified by qPCR for the human β globin gene, as previously described^26^. A standard curve was constructed using QIAGEN EpiTech control human DNA. PCR was carried out in 20 μl reactions containing 5 μl of template or water (negative template control), 1X TaqMan Fast Advanced Master Mix (Applied Biosystems), 0.1 μM each of the forward (5′-GGGCAACGTGCTGGTCTG-3′) and reverse (5′-AGGCAGCCTGCACTGGT-3′) primers, 0.25 μM of probe (5′-FAM-CTGGCCCATCACTTTGGCAAAGAA-BHQ1-3′), and 4.2 μl of water. The PCR program consisted of an initial heating step of 95 °C for 20 s, followed by 40 cycles of 95 °C for 1 s and 60 °C for 20 s. Reactions were performed on a ViiA7 Real-Time PCR System (Life Technologies). All samples and controls were run in duplicate.

Total DNA was quantified using a Qubit high sensitivity dsDNA kit on the Qubit Fluorometer v2.0. Based on the assumption that all non-human DNA in our samples was bacterial, the following equation was used to calculate bacterial DNA concentrations in the cell pellet of each sample: Bacterial DNA (ng/µl) = Total DNA (ng/µl) − Human DNA (ng/µl).

### Bacterial DNA profiling

The full-length 16S rRNA gene was amplified using the primers 27F (5′-AGRGTTYGATYMTGGCTCAG-3′) and 1492R (5′-RGYTACCTTGTTACGACTT-3′), as previously described^27^. These primers were tagged with the universal sequences UNITAG-R (tggatcacttgtgcaagcatcacatcgtag) and UNITAG-F (gcagtcgaacatgtagctgactcaggtcac) (ligated to the 5′end of the primers). A set of three barcoded UNITAG-F and 15 barcoded UNITAG-R primers were designed to generate PacBio sequencing-ready amplicons, using an asymmetric barcoding strategy. PCR was carried out in two rounds. In the first, template was amplified in 50 µl reactions, made up of 7.5 µl template, 0.3 µM each for forward and reverse primers, 1.25 µl each for ArctriZymes dsDNase and DTT, and 13.5 µl of water. Amplification was carried out in a Veriti Thermal Cycler. The program consisted of an initial heating stage of 94 °C for 3 min followed by 35 cycles of 94 °C for 30 s, 55 °C for 30 s, and 72 °C for 2 min. The PCR products were verified by size on a QIAXcel automated electrophoresis system using a DNA high resolution gel cartridge. Amplicons were purified using Macherey–Nagel NucleoMag magnetic beads.

Barcoding of primary amplicons was performed in a secondary amplification procedure. 25 µl reactions were made up of 12.5 µl AccuStart II PCR ToughMix, 20 µM each of the forward and reverse primers, 5 µl of template and 5.5 µl of water. The same cycling conditions were used as in the first amplification, with ten cycles performed. Barcoded samples were pooled in an equimolar concentration and the pool was made up to 50 µl with TE buffer. Finally, the pool was gel purified using the QIAquick Gel Extraction Kit.

The purified, barcoded DNA pool was sent to the Ramaciotti Centre for Genomics, University of NSW, Sydney, Australia where the SMRTbell adapters were ligated onto barcoded sequences and the library was sequenced on a PacBio Sequel system.

PacBio raw reads were demultiplexed to obtain circular consensus sequence (CCS) reads for each sample. CCS reads were filtered to retain only those with a minimum of three full passes and 99.9% sequence accuracy. Raw fastq files were processed using the script GHAP v2.1^28^. GHAP is an amplicon processing pipeline built around tools from USearch^29^ and The Ribosomal Database Project (RDP)^30^, combined with locally-written tools for generating OTU tables. OTU tables were denoised to a minimum of twenty reads.

### Bacterial spike-in assay

To investigate the distribution of an exogenously added bacterial strain within human milk fractions, we performed a spike-in assay to allow quantification of viable bacteria in each fraction. We selected a strain of methicillin resistant *S. aureus* which had been isolated from a breast abscess. Cultures were grown in tryptic soy broth (TSB) at 37 °C. Post-feed whole milk samples (1.5 ml, n = 10) were thawed at room temperature and then inoculated with 3 × 10^5^ CFU of *S. aureus*. The inoculated milk was incubated on a rotary shaker (250 rpm) for 30 min at 37 °C. The fat, pellet, and supernatant fractions were separated by centrifugation (10,000*g* at 4 °C for 6 min) and divided into individual tubes. Fat and pellet fractions were diluted 1:10 in sterile PBS, then enumerated on mannitol salt agar (MSA) plates in triplicate. Supernatants were plated in triplicate without dilution. Blank media was used as a negative control and pure *S. aureus* culture was used as a positive control. Un-inoculated whole milk from each mother served as a further negative control. *S. aureus* colonies were confirmed with coagulase testing.

### Data analysis

Data visualisation and alpha (Shannon) diversity analysis were conducted using MicrobiomeAnalyst^31^. The bacterial profiles of fat and pellet fractions or pre-feed and post-feed samples were compared by PERMANOVA using the software PRIMER-e v7^32^. To account for the within individual structure, mothers were used as a random effect within the PERMANOVA model. To analyse whether any species were differentially abundant in the fat and pellet fractions or in pre-feed and post-feed samples, a univariate linear regression was fit with each species as a response and each sample (fat fraction, cell pellet, pre-feed whole milk, or post-feed whole milk) as the explanatory variable. Comparisons of DNA concentration were performed using student’s t-tests or, if the assumptions were not met, Mann–Whitney tests using R^33^. Given that human milk contains a variable level of fat, the relationship between the percentage fat and the level of *S. aureus* recovered from the fat fraction was assessed by linear regression. Descriptive statistics are reported as mean ± standard deviation. The significant level was set at 0.05.

## Results

### Quantification of total, human, and bacterial DNA

Centrifugation was not able to separate all DNA into the cell pellet; DNA was consistently recovered from the fat fraction of human milk, as assessed by Qubit dsDNA quantification (Table 1). In preliminary testing of four milk samples (unrelated to those used here) DNA was not completely separated to the cell pellet regardless of the centrifugation speed (5000–40,000*g*), with no statistical difference in the percentage of DNA recovered from the fat fraction (*P* = *0*.282). The cell pellet contained a higher concentration of DNA than the fat fraction (*P* = 0.002), with an average of 76.8% (range 61.1–93.8%) of total DNA being recovered from the cell pellet. In five of the six pre/post-feed samples, the post-feed (high fat) sample had a higher concentration of total DNA than the pre-feed (low fat) sample (average of 1.42 and 0.56 ng/µl, respectively; Table 1); however, this difference was not statistically significant (*P* = 0.220). Importantly, inclusion of the fat fraction reduced the efficiency of the DNA extraction. While the post-feed whole milk sample should have theoretically contained the combined total DNA quantity of the fat and pellet fractions, it instead contained significantly less than the cell pellet alone (*P* = 0.047; Table 1). Compared to the cell pellet, inclusion of the fat fraction in the extraction reduced the total yield by an average of 39.4% (range 0–72%).

**Table 1:** Concentration (ng/µl) of total DNA in different human milk fractions by individual (n = 10).

	1	2	3	4	5	6	7	8	9	10	Mean ± SD
Pre-feed whole	0.08		0.75		0.13	0.48		1.81	0.11		0.56 ± 0.67
Post-feed whole	0.39		4.28		0.71	1.78		0.92	0.46		1.42 ± 1.49
Post-feed fat	0.23	0.42	1.73	0.22	0.30	0.81	0.22	0.18	0.28	0.71	0.51 ± 0.48
Post-feed pellet	0.37	1.63	7.56	0.39	1.74	3.23	0.52	3.30	0.59	3.96	2.33 ± 2.27

Measurement of human DNA demonstrated that a considerable proportion (12.4%; range 0.6–20.0%) remained in the fat fraction. This suggests that, while most human cells are able to be pelleted out of human milk by centrifugation, some remain trapped in the fat fraction (difference between cell pellet and fat human DNA concentrations, *P* = 0.011). Given that the human β globin gene is a single copy gene, calculation of human DNA levels presents the opportunity to quantify bacterial DNA in human milk samples without relying on quantification of the 16S rRNA gene, which is variable in copy number and can therefore be only semi-quantitative^34^. On average, the total DNA concentration of the cell pellet was 2.28 ng/µl (range 0.36–7.56 ng/µl), while the average human DNA concentration was 1.16 ng/µl (range 0.18–4.06 ng/µl). Using the assumption that all non-human DNA in human milk is bacterial, the calculated ratio of human to bacteria cells in these extraction eluates is 1.03:1.

Relative bacterial DNA quantification by endpoint 16S rRNA gene PCR showed a similar pattern to that of the total and human DNA. While most bacterial DNA was recovered from the cell pellet (67.3%; range 50.4–91.6%), a substantial portion remained in the fat fraction (32.7%; range 8.4–49.6%) (difference between cell pellet and fat proportions, *P* = 0.002). Together, these results demonstrate that both human and bacterial DNA can remain trapped within the fat fraction of human milk following centrifugation.

### Bacterial DNA profiles of human milk fractions

**Figure 1:**
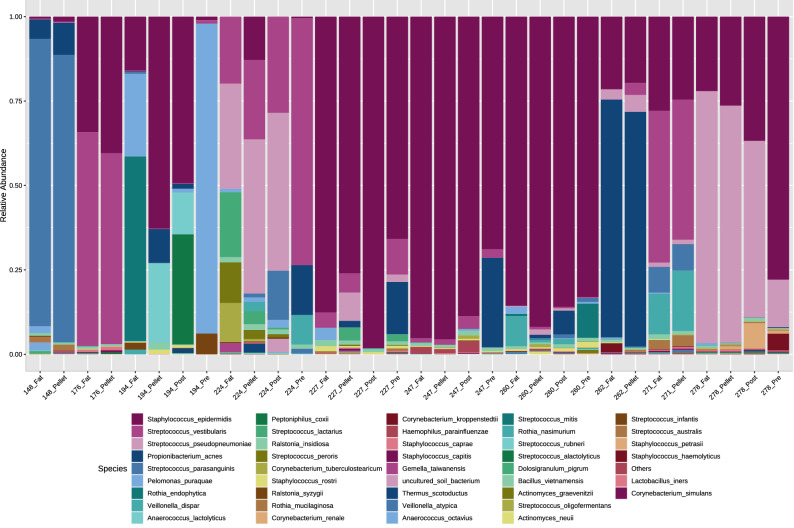
Relative abundance of bacterial species detected in different fractions (fat or cell pellet) of human milk samples (n = 10). For a subset of six mothers, whole milk pre-feed (low fat) and whole milk post-feed (high fat) samples were also available. Data presented here are raw data (i.e. not denoised).

Overall, the DNA profiles of these samples were dominated by reads which mapped to the genera *Streptococcus* and *Staphylococcus*, with a low abundance of anaerobic bacteria, similar to previously reported findings^14,19,35,36^ (Fig. 1) (“Supplementary Information”). There was a high level of inter-individual variation between each mother, with no overall differences between the cell pellet and fat fractions detected (PERMANOVA *P* = 0.926; Shannon diversity *P* = 0.928). The low fat pre-feed samples (3.5 ± 1.9% fat) and high fat post-feed samples (9.5 ± 3.6% fat) did not differ upon PERMANOVA analysis (*P* = 0.68) nor Shannon diversity analysis (*P* = 0.699). Univariate regression analysis revealed two low-abundance species, *Staphylococcus caprae* (*P* = 0.0017) and *Staphylococcus capitis* (*P* = 0.033), to be significantly more abundant in the cell pellet (relative abundance 0.3% and 0.23%, respectively) than in the fat fraction (relative abundance 0.17% and 0.1%, respectively). Further, three low-abundance species (*Anaerococcus octavius*, *Rothia endophytica*, and *Anaerococcus lactolyticus*) were detected in one fraction only. *A. octavius*, was detected in the fat fraction of a single mother’s sample (relative abundance of 1.3%), but not in the corresponding cell pellet. *R. endophytica* was detected in the fat fraction (relative abundance of 3.3%) from one mother’s sample, and in both the fat fraction (relative abundance of 0.1%) and pre-feed sample (relative abundance of 2.1%) from another mother. *A. lactolyticus* was detected in the cell pellet (relative abundance of 4.1%) and post-feed whole milk sample (relative abundance of 2.8%) from a single mother, but not in her corresponding fat fraction or pre-feed sample.

### Bacterial spike-in assay

**Figure 2:**
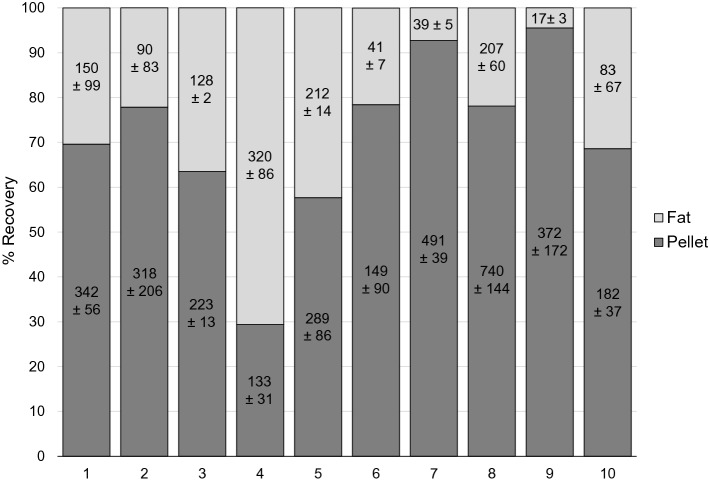
Percent recovery of spiked-in *Staphylococcus aureus* in different human milk fractions from ten samples (different to those used in the microbiome analysis). Percent recovery in supernatant not shown (< 0.03%). Data labels are × 10^4^ CFU/ml ± standard deviation for replicates.

To allow quantification of the distribution of viable bacteria in each human milk fraction, ten post-feed samples (separate from those used for microbiome analysis) were spiked with *S. aureus*. While significantly more of the spiked-in *S. aureus* was recovered from the cell pellet, a substantial proportion (mean = 28.9% recovery) remained in the fat fraction (difference in CFU/ml *P* = 0.020) (Fig. 2). A small quantity of *S. aureus* was recovered from the supernatant for each sample (mean = 0.01% recovery). *S. aureus* was not detected in the control (non-inoculated) milk samples. No relationship was detected between the percentage of fat (11.4%, range 5.4–19%) in each milk sample and the quantity of *S. aureus* recovered from the fat fraction (*P* = 0.549).

## Discussion

The data presented here demonstrate that human and bacterial cells/DNA cannot be completely pelleted out from the fat fraction of human milk. While the majority of cells and DNA are pelleted out by high speed centrifugation (76.8% of total DNA, 87.6% of human DNA, 71.1% of *S. aureus*), a significant quantity remain in the fat fraction. Importantly, bacterial DNA profiles varied between the cell pellet and fat fraction. Two low-abundance *Staphylococcus* species were significantly more abundant in the cell pellet compared to the fat fraction. Further, a number of low-abundance features were detected in only one fraction. However, given that these species were recovered from only one or two mothers’ samples in relative abundances of < 5%, it is difficult to assess whether they represent biological or technical variation. Regardless, the findings presented here are in concordance with similar findings reported in studies of bovine milk^21,22^. These findings have important implications for the interpretations of past studies in this field where the fat layer was discarded prior to DNA extraction^1,8,14–20^, and for the design of future experiments.

Given that the bacterial profiles of the fat fraction and cell pellet varied within individual samples, we next sought to compare pre-feed and post-feed milk samples, which vary substantially in their fat content. We hypothesized that there would be variation in within-feed bacterial DNA profiles based on differences in fat content. However, while pre- and post-feed samples varied in terms of their bacterial DNA profiles, we did not observe any statistically significant patterns. This may be a reflection the low number of samples and the high level of inter-individual variation in milk bacterial communities, or it may suggest that the mechanism through which bacteria become trapped in the fat fraction is mediated by the number, structure and size of the fat globules in the fat fraction. Based on the within-feed analysis, it may be prudent to use a pooled sample from across a feed for microbiome analysis. Pooling of the sample will allow a more global picture of the bacterial microbiome.

For this study, a magnetic bead-based DNA extraction method was used, as the fat in human milk is known to block spin column filters in spin column-based DNA extractions. In spite of this, the inclusion of milk fat interfered with the extraction, as previously observed by others^12^. Inclusion of the fat fraction resulted in a substantial decrease in extraction efficiency of 39.4%. However, it should be noted that this was calculated using post-feed (high fat) samples and that use of pre-feed (low fat) samples may result in less interference with the DNA extraction. This reduction in extraction efficiency is an important consideration given that human milk is already a low biomass sample. A pre-treatment step to dissolve the lipid in human milk may be necessary to overcome this limitation. To date, limited optimisation and validation of DNA extraction methods for human milk exist^1^. Future such work should include extraction and optimization of whole milk samples.

Given the recognised shortcomings of 16S rRNA gene sequencing in terms of absolute quantification of bacteria^34^, a bacterial spike-in assay was performed to trace the distribution of an exogenous bacteria in human milk fractions. On average, 28.9% of the spiked-in *S. aureus* strain was recovered from the fat fraction. Sun et al*.* performed a similar spike-in experiment using three bacterial strains in bovine milk, including a strain of *S. aureus*^21^. These authors reported average recovery rates of 7.4–26.5% in the fat fraction, depending on the strain used. This variation suggests that some bacteria may have a greater affinity for the fat fraction than others. Indeed, the hydrophobicity of *L. reuteri* has been suggested to account for its association with milk fat globules in diary products^37^. Interestingly, we observed a high level of variability in the percentage of *S. aureus* recovered from the fat layer between samples (4.4–70.6%). This suggests that intrinsic properties of the milk, such as milk fat globule size, may determine the extent to which bacteria are trapped within the fat fraction. Binding of bacteria to milk fat globule membrane-associated glycoproteins may also, in part, explain these findings^38^. Collectively, the current evidence suggests that live bacterial cells are trapped by fat in both bovine and human milk. Further working using a range on endogenous milk species is required to assess whether different species display a different affinity for the fat fraction. While quantification of the 16S rRNA gene does not translate to bacterial cell quantification (due to gene copy number variation), other methods such as whole genome sequencing may be useful for more accurately quantifying the bacterial composition in each human milk fraction.

An interesting finding arising from this study is that the ratio of human to microbial DNA in these human milk extraction eluates was approximately 1:1. The use human β globin gene (a single copy human gene) qPCR allows more accurate quantification compared to 16S rRNA gene qPCR. This also allows back calculation of the quantity of bacterial DNA in a sample based on the assumption that all non-human DNA in human milk is derived from bacteria. However, it should be borne in mind that such quantification is reliant on efficient DNA extraction and purification. This ratio may differ with the use of different extraction methods due to variability in the efficiency of lysing human and bacterial cells. While previous studies have measured the cell content of human milk using flow cytometry^39^, to the best of our knowledge we are the first to quantify human DNA in human milk. This finding has implications for whole genome shotgun sequencing studies of human milk, where host DNA depletion may be required prior to sequencing^40^. Additionally, human gDNA can mis-prime with 16S rRNA gene primers/probes and thereby confound low biomass 16S rRNA gene studies^41^. It is, therefore, important to quantify host DNA in low biomass samples, such as human milk, so that appropriate human DNA controls may be used.

Collectively, the results of this study suggest that whole milk should be used for human milk microbiome analyses, with the caveat that DNA extraction efficiency was reduced by 39.4% with the inclusion of the fat fraction.

## Summary

This study has demonstrated that human and bacterial cells and DNA remain trapped in the fat fraction of human milk following centrifugation. Importantly, the bacterial DNA profiles of different human milk fractions vary. These data suggest that, by discarding the fat fraction, researchers may not be identifying the overall bacterial profile of human milk.

## Supplementary Information

Supplementary Tables.

## Data Availability

The raw sequence datasets generated and analysed in this study are available through the Sequence Read Archive under BioProject ID: PRJNA632991.
