# Author Correction: Centrifugation does not remove bacteria from the fat fraction of human milk

**DOI:** 10.1038/s41598-021-88094-x

**Published:** 2021-05-05

**Authors:** Lisa F. Stinson, Jie Ma, Alethea Rea, Michael Dymock, Donna T. Geddes

**Affiliations:** 1 School of Molecular Sciences, The University of Western Australia, Perth, Australia; 2 Mathematics and Statistics, Murdoch University, Perth, Australia; 3 Centre for Applied Statistics, The University of Western Australia, Perth, Australia

Correction to: *Scientific Reports* 10.1038/s41598-020-79793-y, published online 12 January 2021

The original version of this Article contained errors, where P-values resulting from analysis of absolute abundance (number of reads) were mistakenly reported instead of P-values resulting from relative abundance analysis.

As a result, in the Abstract section,

“*Staphylococcus epidermidis* was significantly more abundant in the cell pellet compared to the fat fraction (*P* = 0.038), and three low-abundance species (< 5% relative abundance) were recovered from one fraction only.”

now reads:

“Two low-abundance *Staphylococcus* species (< 0.5% relative abundance) were significantly more abundant in the cell pellet compared to the fat fraction (*P* < 0.04), and three low-abundance species (< 5% relative abundance) were recovered from one fraction only.”

Additionally, in the Results section under the subheading 'Bacterial DNA profiles of human milk fractions',

“There was a high level of inter-individual variation between each mother, with no overall differences between the cell pellet and fat fractions detected (PERMANOVA *P*=0.523; Shannon diversity *P*=0.928). The low fat pre-feed samples (3.5±1.9% fat) and high fat post-feed samples (9.5±3.6% fat) did not differ upon PERMANOVA analysis (*P*=0.563) nor Shannon diversity analysis (*P*=0.699). Univariate regression analysis revealed a single species, *Staphylococcus epidermidis*, to be significantly more abundant in the cell pellet (relative abundance 45.9%) than in the fat fraction (relative abundance 39.8%) (*P*=0.038).”

now reads:

“There was a high level of inter-individual variation between each mother, with no overall differences between the cell pellet and fat fractions detected (PERMANOVA *P* = 0.926; Shannon diversity *P* = 0.928). The low fat pre-feed samples (3.5 ± 1.9% fat) and high fat post-feed samples (9.5 ± 3.6% fat) did not differ upon PERMANOVA analysis (*P* = 0.68) nor Shannon diversity analysis (*P* = 0.699). Univariate regression analysis revealed two low-abundance species, *Staphylococcus caprae* (*P* = 0.0017) and *Staphylococcus capitis* (*P* = 0.033), to be significantly more abundant in the cell pellet (relative abundance 0.3% and 0.23%, respectively) than in the fat fraction (relative abundance 0.17% and 0.1%, respectively).”

Furthermore, in the Discussion section,

“*S. epidermidis*, the most abundant species in these samples, was significantly more abundant in the cell pellet compared to the fat fraction.”

now reads:

“Two low-abundance Staphylococcus species were significantly more abundant in the cell pellet compared to the fat fraction.”

Additionally, the original version of this Article contained a typographical error in the Methods section, under the subheading ‘Bacterial DNA Profiling’.

“OTU tables were denoised to a minimum of ten reads.”

now reads:

“OTU tables were denoised to a minimum of twenty reads.”

Finally, in the original version of the Article the legend for Figure 1 was incomplete.

“Relative abundance of bacterial species detected in different fractions (fat or cell pellet) of human milk samples (n = 10). For a subset of six mothers, whole milk pre-feed (low fat) and whole milk post-feed (high fat) samples were also available.”

now reads:

“Relative abundance of bacterial species detected in different fractions (fat or cell pellet) of human milk samples (n = 10). For a subset of six mothers, whole milk pre-feed (low fat) and whole milk post-feed (high fat) samples were also available. Data presented here are raw data (i.e. not denoised).”

These errors have now been corrected in the HTML and PDF versions of the Article.

