# Supplementary material for: Centrifugation does not remove bacteria from the fat fraction of human milk
Source: Sci Rep. 2021 Jan 12;11:572. doi: 10.1038/s41598-020-79793-y (PMC7804008; doi:10.1038/s41598-020-79793-y)
Supplement: Supplementary file 1 — Supplementary Tables. [file 41598_2020_79793_MOESM1_ESM.pdf]

Centrifugation does not remove bacteria from the fat fraction of human milk

Lisa F. Stinson[1*+], Jie Ma[1*+], Alethea Rea[2], Michael Dymock[3], and Donna T. Geddes[1]

[1] School of Molecular Sciences, The University of Western Australia, Perth, Australia

[2] Mathematics and Statistics, Murdoch University, Perth, Australia

[3] Centre for Applied Statistics, The University of Western Australia, Perth, Australia

Supplementary table 1: Relative abundance of bacterial species in different human milk fractions (fat or cell pellet).

	148		176		194		224		227		247		260		262		271		278	
**Species**	**Fat**	**Pellet**	**Fat**	**Pellet**	**Fat**	**Pellet**	**Fat**	**Pellet**	**Fat**	**Pellet**	**Fat**	**Pellet**	**Fat**	**Pellet**	**Fat**	**Pellet**	**Fat**	**Pellet**	**Fat**	**Pellet**
Acinetobacter lwoffii	0.00	0.00	0.00	0.00	0.00	0.00	0.00	7.95	0.00	0.00	0.00	0.00	0.00	0.00	0.00	0.00	0.00	0.00	0.00	0.00
Anaerococcus lactolyticus	0.00	0.00	0.00	0.00	0.24	24.24	0.00	0.00	0.00	0.00	0.00	0.00	0.00	0.00	0.00	0.00	0.00	0.00	0.00	0.00
Anaerococcus octavius	2.41	0.00	0.00	0.00	0.00	0.00	0.00	0.00	0.00	0.00	0.00	0.00	0.00	0.07	0.00	0.00	0.00	0.00	0.00	0.00
Corynebacterium kroppenstedtii	0.00	0.37	0.00	0.00	0.00	0.00	0.00	0.00	0.00	0.00	0.00	0.00	0.48	0.34	4.98	0.06	0.43	0.57	0.12	0.23
Corynebacterium renale	0.00	0.00	0.00	0.00	0.00	0.00	0.00	0.00	0.00	0.00	0.00	0.00	0.00	0.00	0.00	0.00	0.00	0.00	0.35	0.98
Corynebacterium tuberculostearicum	0.00	0.01	0.05	0.07	0.00	0.00	9.39	0.55	0.00	0.00	0.00	0.23	0.00	0.81	0.00	0.01	0.00	0.00	0.00	0.00
Dolosigranulum pigrum	0.90	0.16	0.00	0.00	0.00	0.00	0.00	0.00	0.00	0.09	0.00	0.00	0.00	0.00	0.00	0.00	0.00	0.00	0.00	0.00
Gemella taiwanensis	0.00	0.52	0.00	0.00	0.00	0.00	2.24	0.00	0.00	0.38	0.00	0.00	0.00	0.27	0.00	0.00	0.04	0.17	0.00	0.00
Haemophilus parainfluenzae	0.00	0.00	0.00	0.00	0.00	0.00	0.00	0.00	0.00	0.00	1.92	1.30	0.00	0.00	0.00	0.00	0.00	0.00	0.00	0.07
Mucilaginibacter daejeonensis	0.00	0.00	0.00	0.00	0.00	0.00	21.95	0.00	0.00	0.00	0.00	0.00	0.00	0.00	0.00	0.00	0.00	0.00	0.00	0.00
Pelomonas puraquae	2.13	0.03	0.05	0.00	24.70	0.00	0.87	1.26	3.56	0.06	0.05	0.00	2.09	0.03	0.57	0.04	0.43	0.03	0.73	0.03
Propionibacterium acnes	5.61	9.33	0.05	0.02	0.24	9.84	0.00	0.00	0.00	1.97	0.00	0.00	0.00	1.02	70.01	70.68	0.00	0.11	0.00	0.03
Ralstonia syzygii	0.28	0.04	0.00	0.02	2.18	0.00	0.22	0.39	0.30	0.03	0.02	0.00	0.08	0.00	0.00	0.04	0.17	0.06	0.15	0.01
Rothia endophytica	0.00	0.00	0.03	0.00	54.96	0.00	0.00	0.00	0.00	0.00	0.00	0.00	0.62	0.17	0.00	0.00	0.00	0.00	0.00	0.00
Rothia mucilaginosa	1.62	1.61	0.00	0.00	0.00	0.00	0.00	0.00	0.00	0.00	0.02	0.00	0.00	0.00	0.00	0.40	2.50	3.26	0.00	0.31
Staphylococcus capitis	0.00	0.05	0.32	0.70	0.00	0.00	0.00	0.16	0.10	0.53	0.09	0.07	0.00	0.07	0.06	0.13	0.34	0.37	0.13	0.21
Staphylococcus caprae	0.04	0.08	0.75	1.05	0.00	0.00	0.00	0.24	0.20	0.31	0.02	0.18	0.05	0.10	0.11	0.21	0.30	0.43	0.25	0.41
Staphylococcus epidermidis	0.60	1.44	34.82	41.13	16.71	64.40	0.00	12.52	90.42	78.06	96.44	96.48	86.90	93.43	20.94	19.47	28.67	25.23	22.35	26.62

Staphylococcus rostri	0.00	0.01	0.13	0.09	0.24	1.29	0.00	0.08	1.28	0.41	0.23	0.23	0.51	0.41	0.23	0.09	0.22	0.14	0.21	0.15
Streptococcus lactarius	0.00	0.00	0.00	0.00	0.00	0.00	14.66	3.39	0.00	3.50	0.00	0.00	0.00	0.00	0.00	0.00	0.00	0.00	0.00	0.00
Streptococcus parasanguinis	86.31	86.04	0.00	0.00	0.48	0.12	0.00	1.10	0.00	0.03	0.05	0.00	0.03	0.30	0.00	0.24	7.83	7.78	0.02	0.03
Streptococcus peroris	0.00	0.00	0.00	0.00	0.00	0.00	9.60	2.44	0.00	0.38	0.00	0.00	0.00	0.00	0.00	0.01	0.00	0.03	0.00	0.00
Streptococcus pseudopneumoniae	0.00	0.09	0.00	0.02	0.00	0.00	25.27	44.41	0.00	8.66	0.00	0.00	0.21	1.63	3.06	5.12	1.29	1.46	75.68	70.92
Streptococcus vestibularis	0.11	0.22	63.79	56.92	0.24	0.12	15.81	22.83	4.15	5.59	1.17	1.52	0.03	0.64	0.06	3.37	45.50	42.13	0.00	0.00
Veillonella dispar	0.00	0.00	0.00	0.00	0.00	0.00	0.00	2.68	0.00	0.00	0.00	0.00	9.00	0.71	0.00	0.12	12.27	18.22	0.00	0.00

Supplementary table 2: Relative abundance of bacterial species in whole milk pre-feed (low fat) and whole milk post-feed (high fat) samples.

Species	194		224		227		247		260		278	
	Pre	Post	Pre	Post	Pre	Post	Pre	Post	Pre	Post	Pre	Post
Actinomyces graevenitzii	0.0	0.0	0.0	0.0	0.0	0.0	0.0	0.0	1.8	0.0	0.0	0.0
Anaerococcus lactolyticus	0.0	9.3	0.0	0.0	0.0	0.0	0.0	0.0	0.0	0.0	0.0	0.0
Anaerococcus prevotii	4.4	0.0	0.0	0.0	0.0	0.0	0.0	0.0	0.0	0.0	0.0	0.0
Anaerococcus tetradius	82.0	0.0	0.0	0.0	0.0	0.0	0.0	0.0	0.0	0.0	0.0	0.0
Corynebacterium kroppenstedtii	0.0	0.0	0.0	0.0	0.0	0.0	0.0	0.0	0.0	0.0	5.0	0.0
Corynebacterium renale	0.0	0.0	0.0	0.0	0.0	0.0	0.0	0.0	0.0	0.0	0.9	7.6
Corynebacterium tuberculostearicum	0.0	0.0	0.0	0.0	0.0	0.0	0.0	0.0	0.0	0.0	0.0	0.0
Haemophilus parainfluenzae	0.0	0.0	0.0	0.0	0.0	0.0	0.2	0.0	0.0	0.0	0.0	0.0
Massilia timonae	0.0	0.0	0.0	3.2	0.0	0.0	0.0	0.0	0.0	0.0	0.0	0.0
Pelomonas puraquae	12.4	0.8	0.0	1.7	0.0	0.0	0.1	0.0	0.6	0.2	0.0	0.0
Peptoniphilus coxii	0.0	52.4	0.0	0.0	0.0	0.0	0.0	0.0	0.0	0.0	0.0	0.0
Propionibacterium acnes	0.0	0.0	0.3	12.9	0.3	0.0	0.3	0.0	0.0	0.2	0.0	0.0
Propionibacterium granulosum	0.0	0.0	0.0	0.0	0.0	0.0	0.0	0.0	0.0	2.1	0.0	0.0
Ralstonia syzygii	0.8	0.2	0.0	0.4	0.1	0.0	0.1	0.0	0.1	0.0	0.0	0.1
Rothia endophytica	0.0	0.0	0.0	0.0	0.0	0.0	0.0	0.0	9.9	0.0	0.0	0.0
Rothia mucilaginosa	0.0	0.0	0.0	0.0	0.8	0.0	0.0	0.0	0.1	0.0	0.4	0.3
Staphylococcus capitis	0.0	0.0	0.0	0.0	0.4	0.0	0.2	0.0	0.0	0.1	0.2	0.3
Staphylococcus caprae	0.0	0.0	0.0	0.0	0.5	0.1	0.1	0.0	0.0	0.2	0.3	0.4
Staphylococcus epidermidis	0.1	36.9	0.6	0.1	79.4	99.1	94.9	0.0	84.5	92.0	78.5	37.4
Staphylococcus rostri	0.0	0.3	0.0	0.0	0.9	0.6	0.5	0.0	1.7	1.0	0.1	0.1
Streptococcus lactarius	0.0	0.0	0.0	0.3	2.4	0.2	0.0	0.0	0.0	0.1	0.0	0.0
Streptococcus mitis	0.0	0.0	0.0	0.1	0.0	0.0	0.0	0.0	0.0	0.1	0.3	0.6
Streptococcus parasanguinis	0.0	0.0	0.0	10.6	0.0	0.1	0.1	0.0	1.2	1.1	0.1	0.0
Streptococcus pseudopneumoniae	0.0	0.0	0.0	33.9	2.7	0.0	0.0	0.0	0.1	0.7	14.1	53.1
Streptococcus vestibularis	0.1	0.1	88.5	20.5	12.5	0.0	3.5	0.0	0.1	0.4	0.0	0.0
uncultured bacterium	0.0	0.0	0.0	1.8	0.0	0.0	0.0	0.0	0.0	0.0	0.0	0.0
uncultured soil bacterium	0.0	0.0	0.0	12.9	0.0	0.0	0.0	0.0	0.0	0.0	0.0	0.0
Veillonella dispar	0.0	0.0	10.5	0.0	0.0	0.0	0.0	0.0	0.0	1.6	0.0	0.0

Wautersiella falsenii	0.0	0.0	0.0	1.8	0.0	0.0	0.0	0.0	0.0	0.0	0.0	0.0
